# Gamified versus non-gamified online educational modules for teaching clinical laboratory medicine to first-year medical students at a large allopathic medical school in the United States

**DOI:** 10.1186/s12909-023-04951-5

**Published:** 2023-12-14

**Authors:** Marie Do, Kimberly Sanford, Susan Roseff, Alexandra Hovaguimian, Henrike Besche, Krisztina Fischer

**Affiliations:** 1 Department of Pathology, Virginia Commonwealth University, 1101 E. Marshall Street, Box 980662, Richmond, VA 23298 USA; 2 Department of Neurology, Beth Israel Deaconess Medical Center (BIDMC), 330 Brookline Avenue, Shapiro 8, Boston, MA 02215 USA; 3 Harvard Medical School, 260 Longwood Avenue TMEC368, Boston, MA 02115 USA; 4 Department of Radiology, Brigham and Women’s Hospital, Harvard Medical School, 260 Longwood Ave, Rm 160, Boston, MA 02115 USA

**Keywords:** Gamification, Medical Education, Laboratory Medicine, Online Preparatory Material

## Abstract

**Background:**

Medical educators seek innovative ways to engage learners efficiently and effectively. Gamification has been explored as one way to accomplish this feat; however, questions remain about which contexts gamification would be most useful. Time constraints and student interest present major barriers for teaching laboratory medicine to students. This study aims to compare two versions of an interactive online module, one gamified and one not, for teaching laboratory medicine concepts to pre-clinical medical students.

**Methods:**

First-year medical students reviewed either a gamified or non-gamified version of an interactive online module in preparation for an in-person flipped classroom session on Laboratory Medicine. Learning theory guided the design of the modules and both contained identical content, objectives, and structure. The “gamified” module included the additional elements of personalization, progress meters, points, badges, and story/role play. After reviewing the module, students completed an anonymous knowledge check and optional survey.

**Results:**

One hundred seventy-one students completed the post module knowledge check as assigned (82 gamified, 89 non-gamified). Knowledge check scores were higher for the students who reviewed the gamified module (*p* < 0.02), corresponding to an effect size of 0.4 for the gamified module. Eighty-one students completed optional post-module surveys (46 gamified, 35 non-gamified). Instructional efficiency was calculated using task difficulty questions and knowledge check scores, and the resulting instructional efficiency was higher for the gamified module. There was no significant difference in the student-reported time required to complete the modules. Additionally, both versions of the module were well received and led to positive ratings related to motivation and confidence. Finally, examination of open-ended survey results suggested that the addition of game elements added value to the gamified module and enhanced engagement and enjoyment.

**Conclusions:**

In this setting, the addition of gamification to an interactive online module enhanced learning outcome, instructional efficiency, student engagement and enjoyment. These results should inspire further exploration of gamification for teaching Laboratory Medicine concepts to pre-clinical medical students.

**Supplementary Information:**

The online version contains supplementary material available at 10.1186/s12909-023-04951-5.

## Background

Today’s medical educators are charged with creating efficient and engaging ways to enlighten their students. Many institutions are reducing the time medical students spend studying basic sciences, while concurrently aiming to foster curious, life-long learners [1]. This changing curricular landscape has led medical educators to adjust their teaching strategies [1–8]. Animations [6], interactive online quizzes [3], adaptive e-learning [7], and virtual reality tours [8] are some of the innovative strategies that have been explored. Importantly, careful curricular planning, with instructional designs grounded in educational theory, and use of comparable comparisons when appropriate are vital for the successful evaluation of new learning activities [1, 9–11]; unfortunately, many educational innovations described in the literature do not meet these standards [2, 6, 12]. For example, in their review of animations used for medical education, Yue et al. found that very few followed the principles of the cognitive theory of multimedia learning [6]. Additionally, McBrien et al. found that fewer than half of the Pathology educational interventions they reviewed described the use of a control group [2].

### Laboratory medicine / clinical pathology instruction

Laboratory medicine, an often overlooked subset of undergraduate pathology education [13], is an area that may benefit from well-planned innovative teaching solutions. As medical knowledge continues to expand, so do discoveries related to diagnostic testing [14, 15]; however, many medical students graduate unprepared to be good stewards of the clinical laboratory [15–20]. Improper utilization of laboratory services can lead to waste, misdiagnosis, and patient harm [14–16, 21, 22]. Consequently, many groups have proposed bolstering students’ exposure to laboratory topics during medical school [13–16, 21, 23]; however, limited time and low student interest present major barriers to this education [24]. Capstone courses [14], case-based interdisciplinary activities [25, 26], and virtual laboratory tours [8, 27] are some of the interventions that have been described to combat these challenges.

### Gamification

Many medical educators have turned to gamification in a bid to innovate and engage students [5, 28–30]. Gamification incorporates game design elements such as points, badges, leaderboards, immediate feedback, and narratives into non-game settings [28, 30]. Importantly, successful gamification must be built upon learning activities with theory-driven, quality designs because, as explained by Landers, “The goal of gamification cannot be to replace instruction, but instead to improve it.” [31]. Additionally, not all game elements appeal to all learners. For example, some learners do not appreciate activities that overemphasize competition or employ extrinsic rewards without a link to learning objectives [29, 30, 32, 33].

Carefully designed gamification shows the potential to improve learner motivation and knowledge [28–31, 33–35], which makes it an attractive tool for Pathology educators who seek to enhance student interest in and understanding of the clinical laboratory. However, medical education is a diverse landscape, and the effects of gamification can vary depending on the context in which is deployed [29, 33, 34, 36]. The use of gamification for teaching medical students about the clinical laboratory is not well established; and, while Tsang et al. recently described a virtual reality lab tour that incorporated gamification in the form of a scavenger hunt activity, no outcome data was reported [8]. Using educational theory and design processes to create and test a gamified activity for teaching clinical laboratory medicine concepts can offer a glimpse into the utility of gamification in this area of undergraduate medical education and inspire future innovation.

### Study aim

The aim of this study was to explore the use of gamification in teaching basic Pathology and Laboratory Medicine concepts to first year medical students at Virginia Commonwealth University School of Medicine by comparing two versions of a carefully designed interactive online module (gamified and non-gamified) to answer the question: Do learning outcomes, instructional efficiency, motivation, confidence, and/or subjective experiences differ for students who review the gamified versus non-gamified module?

## Methods

### Setting and participants

Two versions of a Pathology and Laboratory Medicine module, gamified and non-gamified, were created to provide background information for an in-person flipped classroom session on Laboratory Medicine, part of the first-year curriculum at a large, urban allopathic medical school in the United States. At this institution, first-year medical students enter medical school having already completed a 4-year university degree. Their first year of study consists of basic medical science courses with some early introductory clinical experiences. The discipline of Pathology is introduced to students during the Foundations of Disease course, which typically takes place in December. The Laboratory Medicine session takes place during this course, and offers students an early introduction to the laboratory, an ideal setting to explore ways to foster curiosity and engagement with this topic, as students will have limited structured instruction in this area going forward. There were 188 students enrolled in this course in December 2022.

### Module creation and piloting

Both versions of the module were created using the Articulate 360 Storyline platform (Articulate Storyline 360 [Computer software]. 2022. retrieved from https://articulate.com/360). which allowed for the creation of branching feedback and tracking of points and progress. The modules shared the same objectives and covered the same content, which was reviewed by two Clinical Pathology experts (SR and KS) and was based on the Association of Pathology Chairs suggested goals and objectives for medical students [13]. Additionally, the modules shared the same basic structure, each with the same four sections (Table 1), introductory pretraining, and interactive questions. The shared design was guided by educational theories such as the cognitive load theory [11, 37, 38] and the cognitive theory of multimedia learning [6, 10]. Common to both modules were techniques to manage the intrinsic load (pretraining and segmenting), limit extrinsic load (temporal and spatial contiguity and worked examples) and maximize germane load (interactivity and self-explanation). Strategies to enhance learner motivation such as providing low-stakes assessments with immediate feedback were also employed in both versions of the module. [39].

**Table 1:** Module sections and content (both modules)

Module section	Content covered
What is Pathology?	Defines Pathology as a practice and introduces the different sections of a large academic medical center’s laboratory
Lab Medicine and regulations	Presents the regulations and standards clinical laboratories must follow and relates these requirements to patient results
Phases of lab testing	Explains the different phases of lab testing and identifies common errors in the testing process and when they occur
Choosing the right test	Emphasizes the importance of proper lab utilization and presents the elements to consider before ordering a lab test

**Fig. 1:**
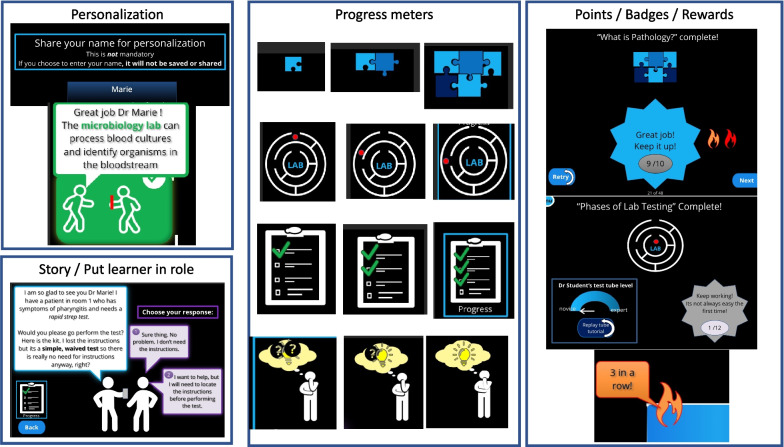
Examples of unique elements added to gamified module. Legend: Personalization, story elements, progress meters and points/badges/rewards were added to the gamified version of the module in an effort to enhance learners’ feelings of engagement, enjoyment, and competence [28, 30]. Personalization included the optional use of the learner's name throughout the module (engagement and enjoyment). Story elements were added by putting learners in a role and asking questions as part of a scenario (engagement and enjoyment). Progress meters offered learners different illustrations to denote progress in different sections (competence and enjoyment). Finally, the incorporation of points, badges and rewards included tracking points earned throughout the module, displaying badges at the end of completed sections, and including extra messages of encouragement when learners achieved multiple correct answers in a row (competence and enjoyement)

Unique elements added to the gamified module included the addition of personalization, progress meters, points, badges, rewards and basic narratives/role play [28, 30] (Fig. 1).

The modules, knowledge check and survey were subject to expert review, cognitive interviews, piloting, and multiple revisions before being implemented in the classroom. During the piloting process, Pathology residents and fourth-year medical students on Pathology rotations were invited to choose one of the modules to review and complete the post-module knowledge check and survey. Seven total knowledge checks (2 gamified and 5 non-gamified) and five surveys (1 gamified and 4 non-gamified) were completed. This exercise allowed for a final check of the logistics of module sharing and data collection.

The knowledge check consisted of 7 multiple choice questions related to the module objectives and was created as a post-module assessment. To bolster content validity, the questions were reviewed by two of the authors (KS and SR), clinical pathology experts. Further, during the piloting phase, it was noted that pathology residents scored higher on average than fourth year medical students (6.9 versus 6.7) on the assessment, an expected finding based on experience level.

Survey questions and, when appropriate, 5-point Likert scale answer choices were constructed using best practices outlined by Artino et al. [40]. Questions related to motivation (interest/relevance and confidence) were inspired by motivational theories and by surveys of student motivation and engagement [39, 41–46]. (The complete survey can be found in the Supplemental material).

### Procedures

An imperfect randomization, utilizing the school’s four pre-existing medical student groups, was employed to assign and evaluate the modules in the full class setting. Assignments were made using a random number generator, with two student groups being assigned the gamified module and two assigned the non-gamified module. Students were encouraged to review their assigned module; however, they were given the option to choose. Additionally, they were able to review both versions of the module and revisit the modules as needed for study after their initial review; however, they were asked to only complete the knowledge check and survey following the first module they reviewed. Sample size was not calculated for this project as the modules were being provided to the given number of students as part of their coursework. All students were assigned the knowledge check, so limiting sample size would not be an option. Also, all voluntary survey responses were valued input for improvement purposes.

In this mixed-methods convergent design, concurrent quantitative and qualitative data were anonymously collected via knowledge checks and surveys embedded at the end of the modules. QuestionPro (QuestionPro survey software [Computer software]. 2023. retrieved from htpps://questionpro.com) was used to create both the knowledge check and survey. Unique module links were crafted so that data from “assigned” and “choice” groups could be sorted while still maintaining anonymity. Completion of the post-module knowledge check was a required component of prework, and these results helped guide the first portion of the subsequent in-person flipped classroom activity, which included an interactive discussion and review of the questions and answers. Therefore, knowledge check assessments submitted after the start of the in-person class period (10:00 AM, 12/5/2022) were not included in the analysis. Survey completion was optional. Neither knowledge check scores nor survey participation affected student course grades.

### Analysis

Quantitative results were analyzed using the statistical software, STATA, Version 17 (StataCorp. 2021. *Stata Statistical Software: Release 17*. College Station, TX: StataCorp LLC.). Statistical significance between scores/ratings for the two module groups was determined using the two-tailed t-test for continuous variables and the Chi-square test for categorical variables, with a *p* value of less than 0.05 considered statistically significant.

Additionally, the effect size (Cohen’s d) was calculated. This calculation expresses the difference between the means of the two groups in units of standard deviation [47]. Cohen’s d was originally described as having three levels of effect sizes (0.2 = small, 0.5 = medium, 0.8 = large) [47]. Importantly, however, an effect size of 0.4 or higher has been used in education as a threshold for identifying “promising” educational interventions [48, 49].

Instructional efficiency, a concept that combines both cognitive effort and learning outcomes, was also calculated. Questions asking students to rate the perceived difficulty of the exercise were included in the survey to help determine the cognitive effort required to complete the activities [50]. For this analysis, the adapted instructional efficiency measure was calculated utilizing standardized Z scores for knowledge check performance and self-perceived difficulty for the two groups [51].

Qualitative results from open-ended survey questions were analyzed by the primary author (MD) utilizing thematic analysis and an inductive approach [52]. A codebook was created, and all responses were coded using this guide (see Supplemental material for codebook). A second coder (KF) checked the codes’ interpretation against the data. Subsequently, categories and themes emerged from iterative examination and analysis.

This project was deemed Educational Quality Improvement during IRB pre-screening at Virginia Commonwealth University and Quality Improvement Program consultation at Harvard University. The SQUIRE 2.0 revised standards for quality improvement reporting excellence were followed [53].

## Results

Overall, 171 students completed the 7-question knowledge check as assigned and on time (Mean (SD) = 6.4 (0.9)). Of these students, the 82 who reviewed the gamified module scored slightly higher on average (Mean (SD) = 6.5 (0.7)) than the 89 who completed the non-gamified module (Mean (SD) = 6.2 (1.0)) This difference was statistically significant (*p* = 0.02), and the effect size was 0.4, making it a “promising” educational intervention [48, 49]. Of note, only 4 students chose the module not assigned (2 gamified and 2 non-gamified).

A total of 81 students chose to fill out the optional post-module survey (35 reviewed the standard, non-gamified module and 46 reviewed the gamified module). Additionally, 55 students answered at least one open-ended survey question (21 reviewed the standard module and 34 reviewed the gamified module). Only one student who completed the survey chose their module (gamified).

No relationship was identified between the self-reported time required to complete the module and the version of module reviewed (*p* = 0.7), with 79 out of the 80 students who answered this question reporting that the module took one hour or less to complete (< 30 min or 30 – 60 min). Additionally, most students reported being only slightly or moderately familiar with the material covered in the module from prior experience or coursework (63/80, 78.8%). Only one student reported being extremely familiar with the material. There was no significant difference in reported familiarity with the material between the students who reviewed the gamified and standard modules (*p* = 0.5).

When asked how easy or difficult the module was to understand, most students for both the gamified and standard module answered “easy” or “neutral” to the question. No student reported that either version of the module was “difficult” or “extremely difficult” to understand. No relationship was identified between the self-perceived difficulty and the version of module reviewed (*p* = 0.6).

**Fig. 2:**
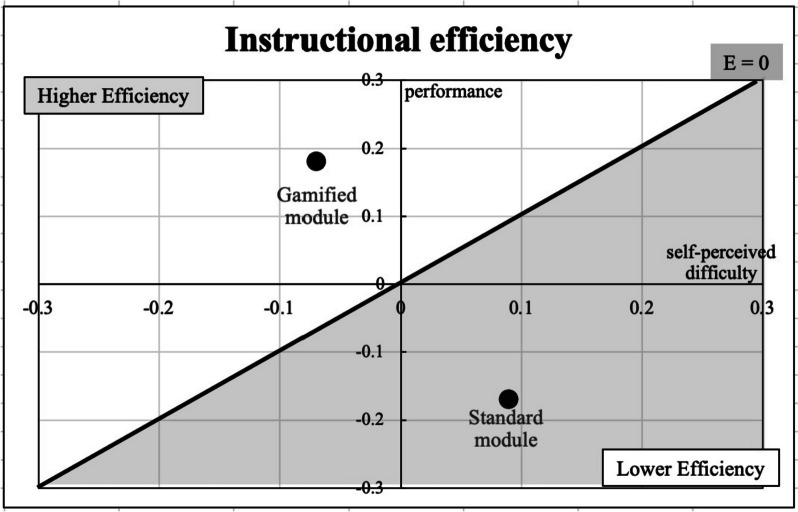
Scatterplot of instructional efficiency of the gamified module and standard module. Legend: Standardized Z scores for knowledge check performance on the y-axis and standardized Z scores for self-perceived difficulty on the x-axis. (efficiency graph format adapted from Paas & Van Merrienboer, 1993 [51])

As can be seen in Fig. 2, instructional efficiency was higher for the gamified module.

### Measures of motivation and confidence

Seven survey questions related to students’ general motivation to learn about lab medicine (relevance, interest, importance, general confidence). The calculated Cronbach’s alpha for this construct was 0.81. The overall results indicate that after completing either version of the module, students were, on average, more than moderately motivated to learn laboratory medicine topics in general (Mean (SD): 3.2(0.6), *n* = 79). Scores for this metric were similar between groups (Mean (SD) = 3.3(0.6), *n* = 45 for gamified group and Mean (SD) = 3.1(0.5), *n* = 34 for standard group), and no statistically significant difference was identified (*p* = 0.3).

An additional set of 6 questions asked students to rate their confidence with the specific material presented in the module. The questions were based on module objectives. The calculated Cronbach’s alpha for this construct was 0.91. The students’ average level of confidence with the specific objectives covered in the Lab Medicine module was 3.2 (SD = 0.7) for all 77 respondents. These results indicate that, on average, the students were more than moderately confident with their understanding of the specific objective-based topics covered in the modules. Average scores for this metric differed slightly between groups, with the students who reviewed the gamified module reporting slightly higher average confidence with these specific topics (Mean (SD) = 3.4 (0.7), *n* = 44) than the students who reviewed the standard module (Mean (SD) = 3.0(0.7), *n* = 33). However, this difference did not reach statistical significance (*p* = 0.06).

### Attitudes and subjective experiences

**Fig. 3:**
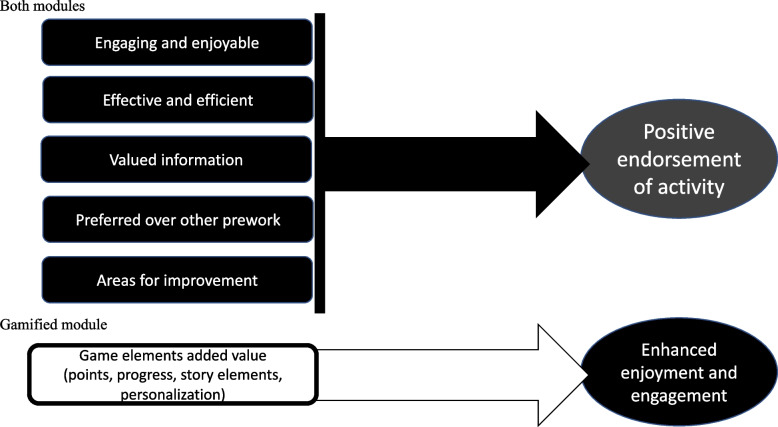
Overview of categories and themes emerging from students’ open-ended survey results. Legend: Review of student comments revealed that both modules were appreciated by students, while the game elements also added value. Students found that both modules conveyed valuable information in an engaging, enjoyable, effective, and efficient manner. Overall, students preferred either module to alternative prework. Feedback for future improvements was similar for both modules and included adding more control for the video portions of the module. The unique elements found in the gamified module added value by boosting student enjoyment and engagement

Of the 81 students who submitted a survey, 55 answered at least one of the open-ended questions asking what they liked about the module, how it could be improved, if they would like to see more modules like this, and for any additional comments. A codebook was created (see Supplemental material for codebook). All responses were coded using this as a guide and categories and themes emerged from further examination. Figure 3 summarizes the themes identified and Table 2 provides illustrative quotes. Students expressed similar positive sentiments towards both versions of the module. Many students expressed enthusiasm for this form of interactive prework**,** and when asked if they would like to see more modules like this in their courses, 89.1% (41 of 46 respondents to this question) answered affirmatively. In their elaboration, many students declared the modules to be superior to other forms of prework, including prereading, reviewing PowerPoint slides, and watching pre-recorded lectures. They appreciated the interactive nature and feedback provided in both versions of the module. Additionally, the students who reviewed the gamified module found that the game elements added value to the experience and enhanced enjoyment and efficiency. Students specifically commented on the progress, personalization and story elements that were unique to the gamified module.

**Table 2:** Themes and representative quotes

**Both modules appreciated by students**
“more engaging than reading, forces students to engage with the material and make connections that may not be obvious to everyone based on reading alone” (standard module)
“I like the interactive nature, much better for retaining information than a straight lecture video.” (gamified module)
“It is a unique way of learning that we don't use everyday, so it's a nice break from traditional lectures, and effective due to its interactiveness.” (standard module)
“This was a really nice alternative to a lecture or a video” (gamified module)
**Game elements added value to gamified module, boosted enjoyment and engagement**
“I liked how interactive it was, and the cartoons showing my progress made it fun.”
“I liked the little guy running to different parts of the lab. I thought the speech bubbles were cute too.”
“I think the gamified aspect to it help it go by a little more quickly”
“I liked how the questions were asked within a conte[x]t/story”

## Discussion

In this project, the use of gamification for teaching Laboratory medicine to first year medical students was explored. To accomplish this, two versions of an interactive online module for teaching Laboratory Medicine to first year medical students at a single institution were evaluated. Both modules were interactive and designed using relevant learning theories as a guide such as the cognitive load theory [11, 37, 38] and the cognitive theory of multimedia learning [6, 10]. One of these modules was “gamified” with the addition of game features (progress meters, points, badges, and story elements). While the gamified module was considered the “intervention,” the standard “control” module was designed to be an effective learning tool on its own. Providing students with a similar alternative learning tool allowed for an effective evaluation of gamification in this setting. Even though the use of gamification has been reported in other areas of medical education, it was important to investigate its use in laboratory medicine education for medical students since data is lacking in this area of study and the effects of gamification can vary depending on the context [34, 36].

Improved learning outcomes were seen for the students who reviewed the gamified module. These students performed better on the post-module knowledge check than the “control” group of students who reviewed the standard (non-gamified module). The difference in scores corresponds to an effect size of 0.4 for the gamified module, suggesting that it is a promising intervention [48, 49].

While the knowledge check findings were encouraging, it was important to consider the instructional efficiency of these modules. This efficiency metric incorporated the students’ ratings of task difficulty in addition to learning outcomes [50, 54]. Using this calculation, the gamified module was more efficient than the standard module at imparting knowledge to students. Additionally, there was no difference in the self-reported time required for module completion or prior familiarity with the material between the two groups.

This instructional efficiency finding indicates that, even though the game elements such as progress indicators, badges, and narrative word bubbles were not essential for learning and may have added to the extraneous load of the module. [11, 37], they did not create a more noticeably difficult learning experience. It is possible that the base module design (shared by both the standard and gamified versions) optimized the cognitive load well enough to allow room in working memory for these additional elements to be added without negative consequences to difficulty or learning. This result highlights the importance of the instructional design component of gamified interventions [30, 31].

Analysis of additional survey results found no significant differences in survey items related to “general motivation for learning lab medicine” or “confidence with specific objective-based laboratory medicine concepts” between the two module types. These findings were surprising since gamification has been shown to boost student motivation in previous studies and this enhanced motivation to learn has been used to help explain improved learning outcomes like those seen in this study [28–31, 33, 34]. Overall, students in both groups reported that they were more than moderately motivated to learn about lab medicine and confident with the material presented. These scores were slightly higher (though not statistically significant) for students who reviewed the gamified module. Perhaps a larger sample or additional survey questions could have provided more insight.

Open ended survey responses suggested that students appreciated the interactivity and feedback included in both versions of the module, while also offering suggestions for improvement such as the desire for increased learner control of the audio/video speed. In addition, the game elements within the gamified module added value to many students’ experiences. The students specifically mentioned liking the progress indicators, personalization, and story-based questions within the gamified version of the module. The students felt these elements created a “fun” and “enjoyable” experience. While not captured in the survey results, this added enjoyment may have promoted student motivation and could help explain the difference in learning outcomes [28, 31].

While this project produced some intriguing results, it is important to note its limitations. First, this was conducted within a single class in a single institution. In addition, while the students were asked to complete the module version assigned to their group, they were also given the allowance to choose their module, which introduced the potential for bias. However, very few students opted to choose (4/171 knowledge check and 1/81 survey). In addition, the students were fully informed of the version of module they were assigned and not all students filled out the optional survey. These two limitations may have also introduced bias. Also, since the students completed this assignment remotely on their own, there may have been differences in their learning environment (i.e., noisy coffee shop versus quiet office) or choices within the module (use of closed captioning option) that could have affected their performance [11, 55]. Additionally, students self-reported the time required for the module completion. Furthermore, learning outcomes focused on immediate testing, and the students’ retention of the information presented in the module was not tested. Also, while the knowledge check was developed and reviewed by experts and piloted with students and residents, the small number of individuals in the piloting phase and the number of questions limited its validity and reliability. In addition, these types of modules were new to the students, so the interest shown towards them by the students may have been due to their novelty [56]. Finally, the gamified intervention included several different game elements (points, badges, progress meters, personalization, narrative). In the present study, it is unknown which of these specific features helped or hindered the learning process.

To address these issues, future studies could include multiple classes and/or institutions, employ strict randomization and blinding, test for knowledge growth and retention, and collect more detailed and objective information about the learners and their time on task. Such studies would produce more statistical power and generalizable results. An experimental design that evaluates the value added by individual game features would also be helpful [49]. In addition, developing and testing a longitudinal series of modules to be used during an entire year or course could reduce the potential for novelty bias [56]. Finally, long term follow up could be considered to not only test retention of facts but also the incorporation of these concepts into medical practice.

## Conclusion

Using gamification to teach Laboratory Medicine concepts in this setting provided students with an engaging and enjoyable experience while also bolstering short-term learning outcomes and instructional efficiency compared to the standard non-gamified control. These results, though localized, should inspire further investigation into the use of this strategy in medical education, particularly in the area of Laboratory Medicine.

### Supplementary Information

**Additional file 1: Supplemental Information. **Survey Questions. **Supplemental material.** Codebook for qualitative analysis.

## Data Availability

The data that support the findings of this study are available from the corresponding author upon reasonable request.
