# Supplementary material for: Gamified versus non-gamified online educational modules for teaching clinical laboratory medicine to first-year medical students at a large allopathic medical school in the United States
Source: BMC Med Educ. 2023 Dec 14;23:959. doi: 10.1186/s12909-023-04951-5 (PMC10720092; doi:10.1186/s12909-023-04951-5)
Supplement: Supplementary file 1 — Additional file 1: Supplemental Information. Survey Questions. Supplemental material. Codebook for qualitative analysis. [file 12909_2023_4951_MOESM1_ESM.docx]

**Supplemental Information:** *Survey Questions*

Post-module survey:

The survey consists of multiple choice and open-ended questions. There are no mandatory questions. Your participation is voluntary and will not affect your grade. This survey is part of a **quality improvement**project looking at motivation and preferences related to the module.

1. How much time did it take you to complete this module?

<30 minutes

30 -60 minutes

61-90 minutes

91-120 minutes

>120 minutes

1. How easy or difficult were the exercises in this module to understand?

Very easy

Easy

Neutral

Difficult

Very difficult

1. How familiar were you with this content from previous coursework?

Not at all familiar

Slightly familiar

Moderately familiar

Quite

familiar

Extremely familiar

1. How important was it for you to prepare for this class?

Not at all important

Slightly important

Moderately important

Quite important

Extremely important

1. How useful did you think this content would be for you when you first looked at it?

Moderately useful

Quite

useful

Extremely useful

Slightly useful

Not at all useful

1. After completing this module, how useful do you think the content of the module will be for you in the future?

Moderately useful

Quite

useful

Extremely useful

Slightly useful

Not at all useful

1. After completing this module, how did your interest in laboratory medicine change?

Much less interested

Slightly less interested

No change in interest

Slightly more interested

Much more interested

1. How interested are you in learning more about laboratory medicine?

Not at all interested

Slightly interested

Moderately interested

Quite interested

Extremely interested

1. How confident are you that completing this module improved your understanding of the clinical laboratory?

Not at all confident

Slightly confident

Moderately confident

Quite confident

Extremely confident

1. How confident are you that completing this module improved your ability to participate in class discussions?

Not at all confident

Slightly confident

Moderately confident

Quite confident

Extremely confident

1. After completing the module, how confident are you in your understanding of the sections of the clinical laboratory?

Not at all confident

Slightly confident

Moderately confident

Quite confident

Extremely confident

1. After completing the module, how confident are you in your understanding of the phases of testing?

Not at all confident

Slightly confident

Moderately confident

Quite confident

Extremely confident

1. After completing the module, how confident are you in your ability to correctly identify common errors and factors that affect patient lab results?

Not at all confident

Slightly confident

Moderately confident

Quite confident

Extremely confident

1. After completing the module, how confident are you in your ability to recognize how the regulations and standards that the laboratory follows relate to patient results?

Not at all confident

Slightly confident

Moderately confident

Quite confident

Extremely confident

1. After completing the module, how confident are you in recognizing the elements to consider when ordering a lab test?

Not at all confident

Slightly confident

Moderately confident

Quite confident

Extremely confident

1. After completing this module, how confident are you in your understanding of the importance of proper lab utilization?

Not at all confident

Slightly confident

Moderately confident

Quite confident

Extremely confident

1. Did you opt to complete the assigned module, or did you choose your module?

chose

assigned

If you opted to choose, which module did you select and why?

What did you like about this module?

How could this module be improved?

Would you like to see more modules like this in your Pathology courses?

Yes No

Why or Why not?

_______________________________________________________________________

Do you have any other comments you would like to share?

**Constructs:**

Efficiency measures (items 1, 2, 3)

General Motivation:

Interest/relevance (items 4, 5, 6, 7, 8)

General confidence (items 9, 10)

Confidence, specific to objectives (items 11, 12, 13, 14, 15, 16)

Supplemental material: *Codebook for qualitative analysis*

| **Number** | **Code Name** | **Definition** | **Illustrative Quotes*** |
| --- | --- | --- | --- |
| **1** | **Helpful questions** | The students find the knowledge checks dispersed throughout the modules helpful | “I liked that there were questions included to test my knowledge.” (M2-20)  “Immediate testing and feedback on the material presented” (M1-24) |
| **2** | **Valued prework** | Students like this option of pre-session preparation more than alternatives | “much better for retaining information than a straight lecture video.” (M2-3) |
| **3** | **Interactive learning** | Students point out the value of active learning found in the modules | “interactive aspect” (M2-44)  “Interactive modules are far more effective than passive lectures.” (M2-40) |
| **4** | **informative** | Students like how helpful the modules are in conveying information | “this was super informative and i loved the interactive portion!” (M2-42) |
| **5** | **fun** | Students describe the enjoyable nature of the activity | “its different, and its more fun to go through” (M2-44) |
| **6** | **engaging** | Students report feeling interested and engaged while completing the activity | “more engaging than reading, forces students to engage with the material” (M1-8) |
| **7** | **gamification** | Students report enjoying elements of gamification | “I appreciated the progress indicators” (M2-26) |
| **8** | **Not preferred** | Student would rather use other methods of learning | “I like PowerPoint learning” (M2-34) |
| **9** | **enthusiasm** | Students express their desire to see additional activities like this with added words and punctuation (more than a “yes” or “no” response) | “Yes, for sure!” (M2-3) |
| **10** | **Efficient** | Students commented on the effectiveness of the modules | “much more efficient & targeted methods than prereading” (M2-18) |
| **11** | **Novelty** | Students appreciated that this activity was different than typical work | “It is a unique way of learning that we don't use everyday, so it's a nice break from traditional lectures” (M1-34)  “it was a nice change from just watching another recorder lecture video” (M2-9) |

* M1 = student reviewed non-gamified module; M2 = student reviewed gamified module
